# A novel microRNAs expression signature for hepatocellular carcinoma diagnosis and prognosis

**DOI:** 10.18632/oncotarget.14452

**Published:** 2017-01-02

**Authors:** Mengxuan Lu, Xia Kong, Huaigao Wang, Guoliang Huang, Caiguo Ye, Zhiwei He

**Affiliations:** ^1^ Sino-American United Cancer Research Institute, Guangdong Medical University, Guangdong Province, China; ^2^ Department of Pathophysiology, Guangdong Medical University, Guangdong Province, China

**Keywords:** microRNA signature, prognosis, diagnosis, TCGA database, HCC

## Abstract

This study aims to identify prognostic microRNAs (miRNAs) biomarkers for diagnosis and survival of hepatocellular carcinoma (HCC) based on large patients cohort analysis. HCC patient cohort data were downloaded from The Cancer Genome Atlas, including paired HCC and adjacent non-cancer tissues. Receiver operating characteristic curve method was used to classify cancer and non-cancer tissues according to microRNAs expression levels. The aberrant microRNAs expression level were ranked and risked for building a prognostic miRNAs signature model. Kaplan–Meier survival was used to analyze the differences among various risk factors in accordance with miRNAs ranking scores. The study showed 33-miRNA signature, 11 were down-regulated and 22 were up-regulated through comparison between cancer samples and non-cancer samples. The maximum correct classification rate is up to 98.7%. Five microRNAs, hsa-mir-3677, hsa-mir-421, hsa-mir-326, hsa-mir-424 and hsa-mir-511-2, significantly correlated with patient survival. The survival rate and time negatively associated with lowering miRNAs index. In the low risk group, over 70% patients showed 5 years survival, while none patients survived longer than 5 years in the high risk group. MiR-424, miR-326 and miR-511 could be applied for HCC diagnostic biomarkers. These five miRNAs were significantly associated with lysosome pathway and D-Glutamine and D-glutamate metabolism pathway via Kyoto Encyclopedia of Genes and Genomes pathway analysis and Gene Ontology annotation. Conclusively, the five miRNAs expression signature could be used as HCC prognostic and diagnostic biomarkers.

## INTRODUCTION

In the past 30 years, liver cancer (mostly hepatocellular carcinoma, HCC) is mainly prevalent mostly in Asia and Africa. However it has become a global disease nowadays [1]. In developing countries, HCC is the second leading cause in male cancer death, while it ranked sixth in more developed countries [2]. Up to now, the early screening of hepatocellular carcinoma mainly depends on liver ultrasound and alpha-fetoprotein (AFP). Liver ultrasound is undoubtedly an economical choice with sensitivity of 60%-90% and specificity of above 90% [3]. Even though serum AFP has been utilized for 40 years with sensitivity of 60%-80% and specificity of 70%- 90%, respectively [4]. It was found that serum AFP concentration was influenced by the tumor size and cancer stage [1]. Moreover, its rise is also commonly seen in chronic liver inflammation and other diseases. Thus its specificity is not satisfying. The European Association for the Study of Liver (EASL) and American Association for the Study of Liver Diseases (AASLD) guidelines do not even recommend AFP as a diadynamic criteria of hepatocellular carcinoma [5]. The AASLD and EASL guidelines only consider the results made from four-phase computed tomography (CT) and dynamic-contrast magnetic resonance (MR), while the Asian Pacific Association for the Study of the Liver (APASL) concerns the size of the lesion [6]. Unfortunately, even if pathological biopsy was deemed as the gold standard, it still has a high false negative rate, regular follow-up inspection is of great necessity [7].

Many studies focused on exploration of cancer diagnosis and prognosis biomarkers using microRNAs (miRNAs, miRs) expression signature. miRNA as biomarker has its advantages, such as stable and high sensitivity. It is reported that detection of miRNAs in section slides was successfully applied [8]. Tissue specific miRNAs are unique identifiers for tumor origin and type [9]. However, the most prominent advantage would be the high-through put sequencing of miRNAs [10]. It has been demonstrated that combination of miR-10b, miR-106b and miR-181a could discriminated HCC patients from normal controls (area under curve (AUC) of 0.85, 0.82, and 0.89, respectively) [11]. Zhang et al. reported that they found serum miR-143 distinguished HCC from healthy individuals with 71% sensitivity and 83% specificit, and miR-215 with 80% sensitivity and 91% sepcificity [12]. Another study identified a panel of 7 miRNAs (miR-122, miR-192, miR-21, miR-223, miR-26a, miR-27a and miR-801) that provided a high diagnostic accuracy of HCC (AUC for training and validation data set are 0.864 and 0.888, respectively) [13].

In this study, we introduced a novel set of miRNAs for HCC diagnosis and prognosis using TCGA database. Because the combined miRNAs signature have more convincing power than every single miRNA, we ranked risk factor for each miRNA, and scored them.

## RESULTS

### Identification of a 33-miRNA signature to discriminate HCC from corresponding noncancerous liver tissues

**Figure 1:**
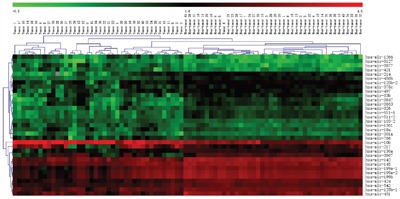
Hierarchical clustering of cancer and non-cancer by 33-miRNA signature Hierarchical clustering of 37 hepatocellular carcinoma samples (left part) and 37 paired non-tumor livers (right part) by the 33-miRNA signature (one hepatocellular carcinoma was misclassified into non-tumor group). The miRNAs expression value showed in the map is Log of the original value downloaded from TCGA miRNAs chip data. Each row represents the expression level of miRNAs, and each column is tissue sample. The color scale setting is according to the MultiExperiment Viewer v4.2 software's indication from -0.5 to 4.8.

miRNAs expression profile of 377 patients were downloaded from TCGA database using TCGA-Assembler [14], specially, 37 paired tumor and non-tumor data were also included. A total of 207 miRNAs were found differently expressed between cancer and non-cancer tissue (student's t test, p<0.05). We got 33-miRNA signature by class prediction and clustering of these paired data using MultiExperiment Viewer v4.2 software. The maximum correct classification rate is up to 98.7% for HCC and noncancerous liver (Figure 1). These 33 miRNAs, in which, 22 were down-regulated and 11 were up-regulated, were listed in Table 1.

**Table 1:** Summary of 33 miRNAs differentially expressed between HCC and non-cancerous liver

microRNA	Tumor (log10, n=37)		Non-tumor (log10, n=37)		Expressionlevel in HCC
	Median	IQR	Median	IQR	
hsa-mir-103-2	0.92	0.21	0.83	0.20	UP
hsa-mir-10b	4.33	0.69	3.23	0.50	UP
hsa-mir-1266	0.78	0.67	0.16	0.43	UP
hsa-mir-1301	0.91	0.48	0.75	0.23	UP
hsa-mir-18a	0.96	0.51	0.93	0.20	UP
hsa-mir-217	2.55	1.99	2.21	0.34	UP
hsa-mir-301a	0.75	0.47	0.64	0.18	UP
hsa-mir-3127	0.51	0.40	0.24	0.32	UP
hsa-mir-3677	0.78	0.50	0.30	0.31	UP
hsa-mir-421	0.75	0.37	0.42	0.31	UP
hsa-mir-766	1.22	0.39	1.08	0.15	UP
hsa-mir-125b-1	2.71	0.48	3.06	0.10	DOWN
hsa-mir-125b-2	1.37	0.43	1.77	0.15	DOWN
hsa-mir-130a	1.49	0.33	2.10	0.14	DOWN
hsa-mir-142	3.02	0.56	3.48	0.21	DOWN
hsa-mir-145	2.86	0.44	3.35	0.24	DOWN
hsa-mir-199a-1	2.29	1.00	3.10	0.25	DOWN
hsa-mir-199a-2	2.54	1.05	3.32	0.21	DOWN
hsa-mir-199b	2.62	1.04	3.36	0.18	DOWN
hsa-mir-214	0.80	0.89	1.55	0.24	DOWN
hsa-mir-326	0.80	0.36	1.08	0.22	DOWN
hsa-mir-33b	0.99	0.65	1.44	0.34	DOWN
hsa-mir-3607	1.58	1.13	2.52	0.49	DOWN
hsa-mir-3647	0.61	0.72	1.16	0.37	DOWN
hsa-mir-3653	0.54	0.74	1.29	0.33	DOWN
hsa-mir-378c	1.10	0.34	1.62	0.23	DOWN
hsa-mir-424	2.13	0.38	2.86	0.30	DOWN
hsa-mir-450b	0.87	0.32	1.45	0.13	DOWN
hsa-mir-497	1.23	0.60	1.55	0.16	DOWN
hsa-mir-511-1	0.75	0.29	1.24	0.19	DOWN
hsa-mir-511-2	0.83	0.36	1.22	0.26	DOWN
hsa-mir-542	2.26	0.30	2.72	0.23	DOWN
hsa-mir-451	2.44	0.55	3.16	0.40	DOWN

### miRNAs signature for HCC prediction

**Figure 2:**
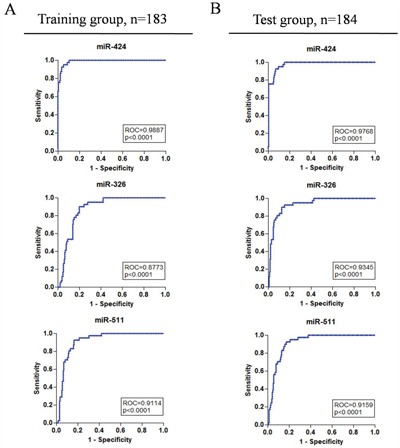
Validation of miRNAs for HCC prediction ROC curve of miRNAs to predicted cancer and non-cancer. The sensitivity and specificity of miR-424, miR-326 and miR-511 were 0.9887, 0.8773 and 0.9114 respectively in the training group (**A.**, n=183, p<0.0001). For validation of the sensitivity and specificity, the test group (n=184) showed miR-424, miR-326 and miR-511 were 0.9768, 0.9345 and 0.9159, respectively (**B.**, p<0.0001).

We randomly divided the TCGA cohort into two groups: training group and test group respectively, using SPSS software. The training group was used to get the area under the ROC curve using ROC method, and the test group was used to validate effect of having or not having cancer outcome. For the above five miRNAs, we got miR-424, miR-326 and miR-511, which sensitivity and specificity were greater than 0.9 (Figure 2A, p<0.0001, sensitivity and specificity were 0.9512 and 0.9029, 0.9024 and 0.8713, 0.9268 and 0.8252). In the test group, The area under the ROC curve were 0.9768, 0.9345 and 0.9159 for miR-424, miR-326 and miR-511 respectively (Figure 2B, p<0.0001). The sensitivity and specificity of miR-125b-2 and miR-451 were greater than 0.85 both in the training group and the test group (Supplementary Figure 2A and 2B, p<0.0001). The signature could be served as a diagnostic marker, and enhanced the accuracy when combined with pathological diagnosis.

### Identification of five miRNAs associated with HCC patients survival

**Figure 3:**
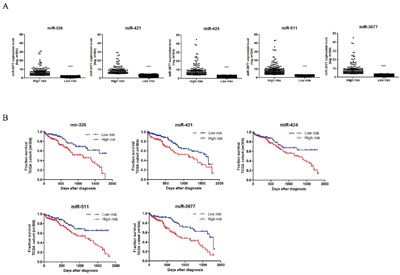
Five miRNAs were associated with overall survival of HCC patients Expression level values were log transformed and represent the average expression between ROC curve discrimination in 304 patients **A.** Kaplan–Meier survival analysis to evaluate the 5 miRNAs prognostic effects. Patients were stratified into the low risk group or high risk group based on overall survival rate (**B.**,***, represents p<0.0001, significance was determined using the log-rank test).

In order to identify survival sensitive miRNAs profile, we used ROC curve to discriminate the 33 miRNAs in 304 patients. These patients have completed miRNAs data and clinical data. Among the 33 miRNAs, 8 miRNAs including hsa-mir-3677, hsa-mir-421, hsa-mir-125b-2, hsa-mir-326, hsa-mir-424, hsa-mir-511-1, hsa-mir-511-2 and hsa-mir-451, showed significantly different outcome after ROC curve analysis. We dichotomized 304 patients according to the miRNAs value comparison to ROC cutoff score, and named high level or low level group. The 8 miRNAs with patients survival time were analyzed Kaplan–Meier survival analysis. Only hsa-mir-3677, hsa-mir-421, hsa-mir-326, hsa-mir-424 and hsa-mir-511-2, were significantly correlated with patients survival (Figure 3, p<0.05, Log-rank test). The shorter time group was named high risk group, and the longer time as low risk group.

### Prognostic five miRNAs signature index for HCC survival analysis

**Figure 4:**
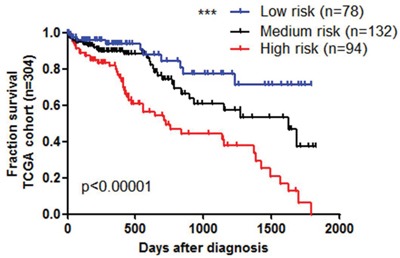
Prognostic biomarker for HCC using five miRNA signature Kaplan–Meier survival analysis to evaluate the prognostic 5 miRNAs signature index models (A). High risk index is above 4 score, medium risk 2-3 score, and low risk below 2 score.

In the procedure of miRNAs signature index definition, we defined high risk group patients getting 1 score, while low risk group getting 0 score for each miRNA. Under this criteria, the highest score would be 5, and the lowest score was 0. We ranked 304 HCC patients according to the miRNAs signature index, and grouped them into 3 groups. High risk group represented miRNAs signature index was above 4 scores, and medium risk group was 2-3 scores, when low risk group was below 2 scores. Kaplan–Meier survival analysis showed these 3 groups are significantly correlated with patients survival (Figure 4). In the low risk group, over 70% patients showed 5 years survival, while none patient survived longer than 5 years in the high risk group (Figure 4, blue line as low risk, red line as high risk). In the result, we could also conclude that the survival rate and time increase companied with lowed miRNAs signature index. MiR-3677 and other miRNAs showed less sensitivity and specificity in cancer prediction (less than 0.7, data not shown)

### Kyoto encyclopedia of genes and genomes (KEGG) signal pathway and gene ontology (GO) annotation of five miRNAs predicted genes

We used KEGG pathway to analyze the 5-miRNA potentially down- or up-regulated genes in light of their pivotal role in patients survival prognosis and disease diagnosis. KEGG pathway was usually facilitated to illustrate all the associated pathways containing differentially expressed genes. Interestingly, only four significant pathways were shown even the p-value was larger than 0.05 (Table 2), including lysosome pathway and glutamate metabolism pathways. In Go annotation, three main classes of processes were distinguished according to the ordering of the ontology system. They are biological processes, cellular components and molecular function. In this study, we defined the significantly process with p-value less than 0.05. In molecular functions, 19 processes were exhibited to be associated with 5-miRNA predicted genes (Table 3). In the table, many processes were involved in membrane protein transporter and enzyme activity function, especially, glutamate transmembrane transporter activity and dehydrogenase activity. Over 34 processes were significant shown in the GO term biological process (Supplementary Table 1). The four most genes involved processes were membrane transporter, organic development and function associated, cell growth and ubiquitination. In cellular components, endomembrane system and plasma membrane part consisted of the main process (Supplementary Table 2, GO:0012505 and GO:0044459).

**Table 2:** KEGG pathway analysis of 5-miRNA potential regulated genes

Pathway	Count	Genes	p-value
Lysosome	7	AP1M2, CTSD, CTSC, CTSS, CTNS, CLTC, GGA3	0.0161
Nitrogen metabolism	3	CA14, GLUD2, GLUD1	0.0604
D-Glutamine and D-glutamate metabolism	2	GLUD2, GLUD1	0.0682
Insulin signaling pathway	6	PRKAB2, PRKCI, GYS1, MKNK1, PPARGC1A, AKT3	0.0868

**Table 3:** GO term of molecular function analysis of 5-miRNA potential regulated genes

GO Term	Count	Genes	p-value
GO:0015179 L-amino acid transmembrane transporter activity	6	SLC36A1, SLC17A8, SLC1A2, SLC1A3, CTNS, SLC25A15	0.0011
GO:0015171 amino acid transmembrane transporter activity	7	SLC36A1, SLC17A8, SLC1A2, SLC1A3, PDPN, CTNS, SLC25A15	0.0014
GO:0005275 amine transmembrane transporter activity	7	SLC36A1, SLC17A8, SLC1A2, SLC1A3, PDPN, CTNS, SLC25A15	0.0046
GO:0000166 nucleotide binding	63	ABCF1, ADCY1, SEPHS1, RBM15B, STK35, LEMD3, RBM6, TPK1, ACTR3, ACTR2, ANKRD17, PAK2, AAK1, DHX33, TLK2, EIF2B2, ARL5B, AKT3, RBM12, RHOH, RAP2B, ARL1, KIF5C, PRKCI, PPARGC1A, RAD50, MARK1, RND3, ACVR2A, KIF1B, RFK, TESK2, STEAP2, RAB10, SLC27A4, GLUD2, GLUD1, MKNK1, IGF2BP3, EPHB4, MAP3K2, CHD2, PPIL4, MSI2, IDH1, DCLK2, HCN4, POLQ, MYO5B, RAB2A, DNM1L, ELAVL2, ELAVL3, DOCK8, RAB33B, SIRT3, MEF2D, HSPA12B, PLK2, ILF2, CDC42BPA, MERTK, SMC1B	0.0123
GO:0030674 protein binding, bridging	7	KHDRBS1, COL19A1, VAV3, GRAP, ABI2, RAD50, TOB1	0.0143
GO:0005070 SH3/SH2 adaptor activity	5	KHDRBS1, VAV3, GRAP, ABI2, TOB1	0.0193
GO:0005313 L-glutamate transmembrane transporter activity	3	SLC17A8, SLC1A2, SLC1A3	0.0214
GO:0015172 acidic amino acid transmembrane transporter activity	3	SLC17A8, SLC1A2, SLC1A3	0.0253
GO:0015296 anion:cation symporter activity	4	SLC1A2, SLC1A3, SLC12A2, SLC20A2	0.0268
GO:0004842 ubiquitin-protein ligase activity	8	RNF8, RNF144B, WWP1, UBE4B, RNF217, FBXW2, FBXO10, FBXL2	0.0357
GO:0004352 glutamate dehydrogenase activity	2	GLUD2, GLUD1	0.0416
GO:0005314 high-affinity glutamate transmembrane transporter activity	2	SLC1A2, SLC1A3	0.0416
GO:0070728 leucine binding	2	GLUD2, GLUD1	0.0416
GO:0004353 glutamate dehydrogenase [NAD(P)+] activity	2	GLUD2, GLUD1	0.0416
GO:0016639 oxidoreductase activity, acting on the CH-NH2 group of donors, NAD or NADP as acceptor	2	GLUD2, GLUD1	0.0416
GO:0003755 peptidyl-prolyl cis-trans isomerase activity	4	PPIF, FKBP8, FKBP5, PPIL4	0.0423
GO:0005310 dicarboxylic acid transmembrane transporter activity	3	SLC1A2, SLC1A3, SLC25A10	0.0435
GO:0016859 cis-trans isomerase activity	4	PPIF, FKBP8, FKBP5, PPIL4	0.0482

When combined the analyses of KEGG and GO annotation, we considered that the most significantly associated pathway was lysosome pathway, because it is tightly linked to cell metabolism and dead cell clearance, and to organ development, cell growth and ubiquitination in GO term. Another appealing pathway was the D-Glutamine and D-glutamate metabolism pathway in KEGG analysis. Many processes and functions indicated that membrane transporters were pivotal in GO term, which might be involved in glutamine-axis function. However, the map of KEGG and GO annotation is too large to get a very specific result or to draw a convinced conclusion. The axis of miRNA-targets-function-disease in HCC diagnosis and prognosis needs to be deeply studied.

## DISCUSSION

miRNAs are promising biomarkers for cancer diagnosis and prognosis. Increasing studies on miRNAs as biomarkers have been reported. Wei et al. conducted a microarray consisting of 683 miRNAs used in profiling 166 hepatocellular carcinomas in South China, and found 4 miRNAs (hsa-mir-451, hsa-mir-766, hsa-mir-103 and hsa-mir-18a) were potential biomarkers [15]. In another study, an integrated miRNA signature was identified from 26 published HCC datasets, and validated by TCGA database. Three miRNAs were the same with our findings: hsa-mir-214, hsa-mir-145 and hsa-mir-199a [16]. In our study, a 33-microRNA signature was identified to discriminate hepatocellular carcinoma from corresponding noncancerous liver tissues, among which 5 miRNAs (hsa-mir-3677, hsa-mir-421, hsa-mir-326, hsa-mir-424 and hsa-mir-511-2) were identified significantly associated with patients survival. In addition, five miRNAs (miR-424, miR-326, miR-511, miR-125b-2 and miR-451) were identified to provide high diagnostic accuracy of HCC. The main reason might be probably that miRNAs expression profiles varied considerably in different studies due to different technological platforms and sample conditions, like stage and pathological grading.

The miRNAs panel also differentiated HCC from the healthy (AUC 0.941), chronic hepatitis B (AUC 0.842), and cirrhosis (AUC0.884), respectively [13]. MiRNA-21 ROC analysis showed an AUC of 0.773 with 61.1% sensitivity and 83.3% specificity when differentiating HCC from chronic hepatitis [17]. The combination of miR-16, AFP, AFP-L3 and DCP correctly identified HCC from healthy controls up to 92.4% sensitivity and 78.5% specificity [18]. To our disappointment, we did not find the association between miRNAs signature and HBV/HCV, or AFP, or other routine biomarkers. It suggested that there are some differences between the western and eastern countries in terms of tumorigenesis and tumor progression of HCC. Chinese patients are mainly infected with HBV whereas most US patients usually carry HCV instead.

Moreover, we have taken Go annotation into consideration. The result of GO annotation showed glutamate transmembrane transporter activity and dehydrogenase activity were the most related cellular components and molecular function. This further confirmed our conclusion. In KEGG pathway analysis of five miRNAs potential targets, several genes were reported to increase HCC development. Lysosome pathway makes organelle and protein homeostasis and acts as a cell survival mechanism under a variety of stress conditions [19]. AKT/mTOR activates autophagic lysosome pathway, which is regarded as autophage or apoptosis switch in injured liver cell fate [19]. It is reported impaired lysosomal maturation may be crucial to the carcinogenesis of HBV-related HCC [20]. Autophagy is an important mediator for the suppression of liver tumorigenesis. Its deficiency is associated with a poor prognosis of HCC [21]. The HCC development was also associated with expression of early HCC markers (glutamine synthetase, glypican 3, heat shock protein 70, and the serum marker AFP) [22–24]. The neoplastic nature of the HCC was confirmed by histology and expression of the HCC marker glutamine synthetase (GS) [25]. GS is the target of the Wnt/beta-catenin pathway in the liver, therefore, glutamine metabolism by beta-catenin is a contributing factor to HCC development [26].

Several miRNAs of our signatures have not been reported previously in HCC, which may provide a novel molecular approach for HCC diagnosis and prognosis. Our 33-microRNA signature was also essential for identifying potential targets for HCC therapy and monitoring the tumor progression and recurrence. As HCC is a highly complex, multi-factorial and heterogeneous disease, many miRNAs are dysregulated during tumorigenesis and progression. Therefore, a combination of multiple circulating miRNAs or a plasma/serum miRNA panel could provide more accurate information than just one single miRNA for the diagnosis and prognosis of HCC. Moreover, the combination of serum/plasma miRNAs with already established markers (such as AFP, FP-L3 and DCP etc.) may also improve the performance of HCC diagnosis [27]. In spite of a great amount of evidence for the presence of circulating miRNAs, their functions and mechanisms have not thoroughly been clarified yet. We have to agree that there are several limitations in the study design and the findings. The pivotal limit of the study design is the lack of cross-validation with different HCC patient cohort. Such cohorts could be from other database, or our collected patient data. After cross-validation with other cohort, the miRNAs signature for diagnosis and prognosis will be more convincing. Secondly, the finding of tissue miRNAs signature limits the use of early diagnosis for HCC detection, unless such signature is tested in serum samples. Actually, we are building up a database related to HCC patient which belongs to our cancer institute. The data includes gene expression array, miRNA expression profile, gene promoter methylation array and clinical laboratorial markers from serum or tissues. We look forward to solving the above limitations after the accomplishment of the database.

## MATERIALS AND METHODS

### Patients selection

The results shown here are wholly based upon data generated by The Cancer Genome Atlas (TCGA) Research Network:https://gdc.cancer.gov/. The expression profile of 1042 miRNAs was from 377 patients who were diagnosed of HCC and 37 normal controls. Thirty-seven paired tumor and non-tumor tissues data were also included. Due to the missing follow-up survival data, we excluded those samples which survival status was not recorded. Actually, 334 samples were used for further analysis. The flowchart of the study was shown in Supplementary Figure 1.

### Setting cutoff score based on ROC curve

We used the maximum value of the sum of specificity and sensitivity as a cut-off point for each miRNAs [28, 29]. For each miRNAs, two groups were separated, and named as high level or low level group. Kaplan–Meier survival analysis was employed to evaluate the 8 miRNAs’ correlation with patients survival.

### Prognosis prediction using miRNAs scoring

In the survival analysis, patients were stratified into the low risk group and high risk group based on their expression level of the 5 miRNAs which were significantly correlated with patients survival. Higher level was considered as high risk group, lower level as lower risk group. We defined high risk group patients getting 1 score, while low risk group getting 0 score for each miRNA. Therefore, the highest score would be 5, and the lowest score was 0.

### Statistics

Statistical analyses were used Statistical Package for the Social Science (SPSS) 13.0, except for the hierarchical clustering analysis (HCL) was conducted by MultiExperiment Viewer version 4.2. Student's t test was applied to analyze the differentially expressed miRNAs. Receiver operating characteristic (ROC) analysis was used to discriminate 8 miRNAs which were significantly different among the 33 miRNAs, and to classify HCC and noncancerous liver samples. Kaplan–Meier survival analysis was employed to evaluate the correlation of 8 miRNAs with patients survival. All p<0.05 were marked as significantly different.

## SUPPLEMENTARY MATERIALS FIGURES AND TABLES





## Abbreviations

miRNA: microRNAs
HCC: Hepatocellular Carcinoma
TCGA: the Cancer Genome Altas
AFP: alpha-fetoprotein
ROC: Receiver Operator Characteristic
AUC: Area Under Curve
EASL: The European Association for the Study of Liver
AASLD: American Association for the Study of Liver Diseases
KEGG: Kyoto Encyclopedia of Genes and Genomes
GO: Gene Ontology
