# Supplementary material for: A novel microRNAs expression signature for hepatocellular carcinoma diagnosis and prognosis
Source: Oncotarget. 2017 Jan 2;8(5):8775–84. doi: 10.18632/oncotarget.14452 (PMC5352440; doi:10.18632/oncotarget.14452)
Supplement: Supplementary file 1 [file oncotarget-08-8775-s001.pdf]

A novel microRNAs expression signature for hepatocellular carcinoma diagnosis and prognosis

SUPPLEMENTARY FIGURES AND TABLES

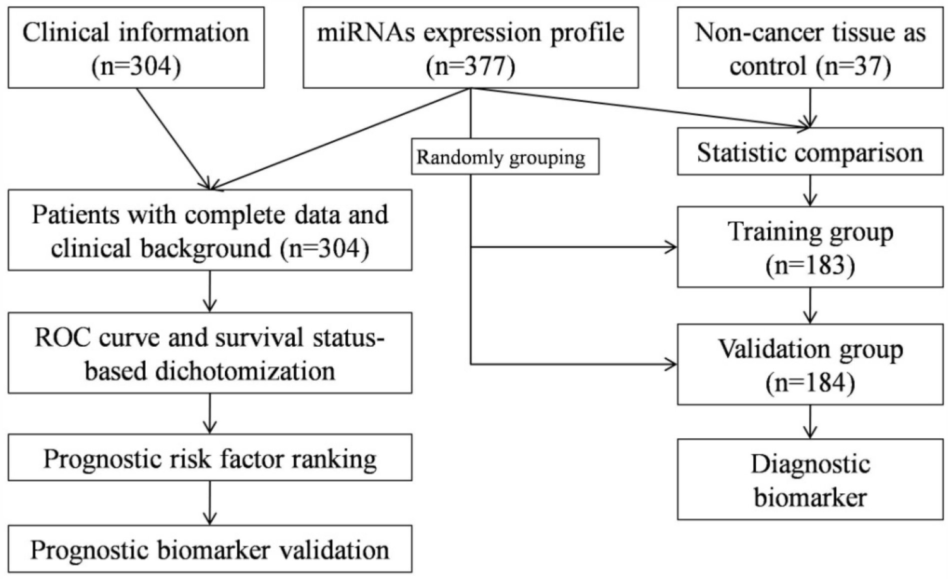

Supplementary Figure 1: Flowchart of the study.

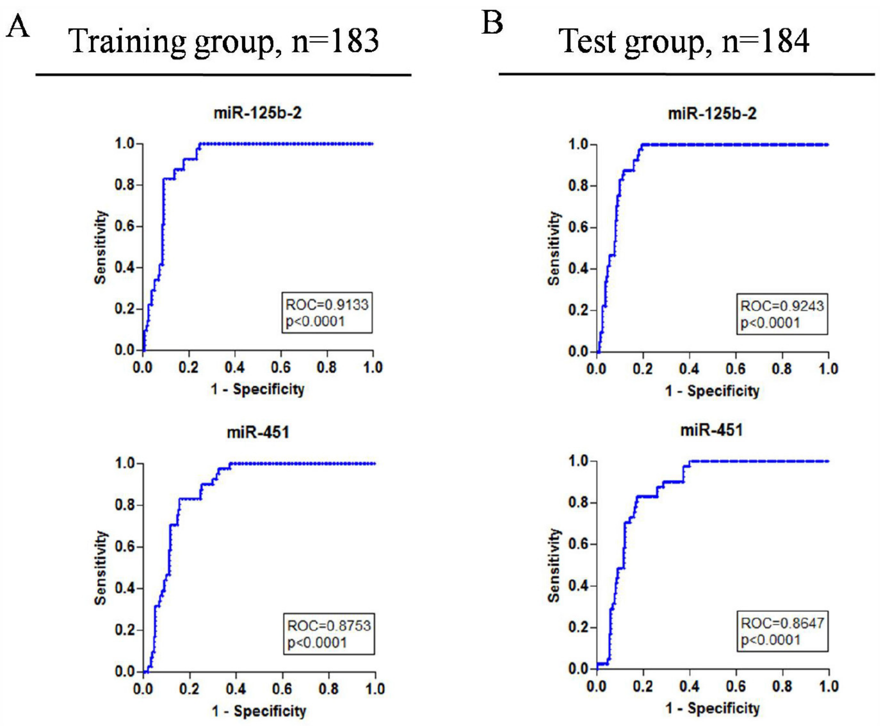

Supplementary Figure 2: Validation of miRNAs for HCC prediction. The sensitivity and specificity of miR-125b-2 and miR-451 was 0.9133 and 0.8753 respectively (**A.**, n=183, p<0.0001). The test group showed ROC value was 0.9243 and 0.8647 respectively (**B.**, n=184, p<0.0001).

Supplementary Table 1: GO Term of Biological Process

See Supplementary File 1

Supplementary Table 2: GO Term of Cellular Component

GO Term	Count	Genes	p-value
GO:0005875 microtubule associated complex	7	KIF1B, ATG4B, KIF5C, KLC4, MAP7, DCTN6, DCX	0.0155
GO:0044459 plasma membrane part	58	ABCF1, SLC22A12, SLC20A2, TGFB3, SYT9, ZFYVE20, CLTC, MMP24, KCNJ13, ACTR3, SLC1A2, SLC1A3, SPRED2, TOMM34, RHOH, RAP2B, ARL1, ARC, PDPN, MDGA1, PRKCI, STIM1, IGSF6, IGSF5, ACVR2A, CD80, GRM8, EPB41L5, CLDN2, LAMC2, HAS3, EDA, RAB10, EIF5A2, SNX18, FKBP8, AP1M2, PANX1, DRD2, FCER2, ABI2, CDH2, EPHB4, RIMS4, SYPL1, CD69, PRIMA1, TES, GABRA4, SLC12A2, NCKAP1, GJB2, SLC17A8, CDC42BPA, TGFBR3, MAP7, MERTK, SLC25A17	0.0161
GO:0031252 cell leading edge	8	ACTR3, SLC1A2, CTTN, PDPN, CDC42BPA, ABI2, CDH2, FBXO10	0.0195
GO:0012505 endomembrane system	25	FKBP8, SLC9A6, AP1M2, LEMD3, DSCR3, CLTC, SSR1, ACTR3, AAK1, NUP50, CYP26B1, FNDC3A, SPTLC2, STIM1, GJB2, MAN2A2, APOL2, SLC17A8, KIF1B, CHST6, EDA, CYP8B1, STEAP2, EIF5A2, LRPPRC	0.0207
GO:0009898 internal side of plasma membrane	13	RAP2B, ARL1, SNX18, FKBP8, AP1M2, SPRED2, CLTC, ZFYVE20, RAB10, EIF5A2, NCKAP1, RHOH, TOMM34	0.0230
GO:0031090 organelle membrane	32	FKBP8, COX11, AP1M2, SLC9A6, LEMD3, CLTC, SSR1, ACTR3, SLC1A3, CYP26B1, PXMP4, FNDC3A, TOMM34, DNM1L, SPTLC2, STIM1, VTI1B, GJB2, MAN2A2, APOL2, PPIF, SLC17A8, KIF1B, CHST6, SLC25A10, CYP8B1, STEAP2, CTNS, EDA, SLC25A15, LRPPRC, SLC25A17	0.0260
GO:0044431 Golgi apparatus part	12	ACTR3, MAN2A2, STX6, ARL1, DNM1L, AP1M2, CHST6, CLTC, STEAP2, GOLGA4, GGA3, GJB2	0.0315
GO:0000151 ubiquitin ligase complex	6	RNF8, CUL3, RNF144B, WWP1, UBE4B, FBXO10	0.0325
GO:0042995 cell projection	22	ARC, PANX1, SLC22A12, PDPN, DRD2, KIF5C, LDB3, ABI2, CDH2, NCKAP1, ACTR3, ACTR2, SLC1A2, CTTN, SLC1A3, GRM8, LRRTM1, CNTN4, SEMA3A, MERTK, HCN4, FBXO10	0.0350
GO:0031012 extracellular matrix	13	MATN2, ADAMTS18, ADAMTS19, TGFB3, OLFML2A, MMP24, ADAMTS6, COL19A1, SLC1A3, COL12A1, LAMC2, NCAN, ADAMTS5	0.0402
GO:0030027 lamellipodium	5	ACTR3, CTTN, PDPN, ABI2, CDH2	0.0492
