# Supplementary material for: A novel microRNAs expression signature for hepatocellular carcinoma diagnosis and prognosis
Source: Oncotarget. 2017 Jan 2;8(5):8775–84. doi: 10.18632/oncotarget.14452 (PMC5352440; doi:10.18632/oncotarget.14452)
Supplement: Supplementary file 2 [file oncotarget-08-8775-s002.docx]

**Supplemental table s1. GO Term of Biological Process**

| **GO Term** | **Count** | **Genes** | **p-value** |
| --- | --- | --- | --- |
| GO:0030510 regulation of BMP signaling pathway | 7 | ACVR2A, FKBP8, SOST, HOXA13, SOSTDC1, LEMD3, TOB1 | 0.0000 |
| GO:0015849 organic acid transport | 11 | SLC36A1, SLC17A8, SLC1A2, SLC1A3, SLC22A12, PDPN, DRD2, SLC25A10, CTNS, SLC25A15, SLC27A4 | 0.0012 |
| GO:0046907 intracellular transport | 26 | RAB2A, STX6, ARL1, AP1M2, RBM15B, PRKCI, TGFB3, DSCR3, VTI1B, ICMT, STXBP4, CLTC, ZFYVE20, SSR1, PPIF, KIF1B, SLC1A3, SLC25A10, NUP50, EXOC5, STEAP2, SLC25A15, LRPPRC, GGA3, TOMM34, TOB1 | 0.0034 |
| GO:0046942 carboxylic acid transport | 10 | SLC36A1, SLC17A8, SLC1A2, SLC1A3, PDPN, DRD2, SLC25A10, CTNS, SLC25A15, SLC27A4 | 0.0041 |
| GO:0001889 liver development | 6 | CCND1, JARID2, CEBPG, ONECUT2, TGFBR3, ICMT | 0.0052 |
| GO:0045137 development of primary sexual characteristics | 9 | ACVR2A, CCND1, FGF7, HOXA13, DMRT1, IDH1, EIF2B2, FNDC3A, GJB2 | 0.0057 |
| GO:0006537 glutamate biosynthetic process | 3 | SLC1A3, GLUD2, GLUD1 | 0.0064 |
| GO:0009968 negative regulation of signal transduction | 12 | DAND5, CCND1, SOST, DRD2, SOSTDC1, CYP26B1, ONECUT2, TGFB3, LEMD3, AXIN2, TOB1, RHOH | 0.0076 |
| GO:0030514 negative regulation of BMP signaling pathway | 4 | SOST, SOSTDC1, LEMD3, TOB1 | 0.0095 |
| GO:0046546 development of primary male sexual characteristics | 6 | ACVR2A, CCND1, HOXA13, DMRT1, FNDC3A, GJB2 | 0.0122 |
| GO:0006865 amino acid transport | 7 | SLC36A1, SLC17A8, SLC1A2, SLC1A3, PDPN, CTNS, SLC25A15 | 0.0122 |
| GO:0015807 L-amino acid transport | 4 | SLC36A1, SLC1A2, SLC1A3, CTNS | 0.0122 |
| GO:0007548 sex differentiation | 9 | ACVR2A, CCND1, FGF7, HOXA13, DMRT1, IDH1, EIF2B2, FNDC3A, GJB2 | 0.0152 |
| GO:0010648 negative regulation of cell communication | 12 | DAND5, CCND1, SOST, DRD2, SOSTDC1, CYP26B1, ONECUT2, TGFB3, LEMD3, AXIN2, TOB1, RHOH | 0.0169 |
| GO:0048608 reproductive structure development | 8 | ACVR2A, CCND1, FGF7, HOXA13, DMRT1, IDH1, EIF2B2, FNDC3A | 0.0179 |
| GO:0046661 male sex differentiation | 6 | ACVR2A, CCND1, HOXA13, DMRT1, FNDC3A, GJB2 | 0.0193 |
| GO:0048193 Golgi vesicle transport | 8 | RAB2A, STX6, ARL1, AP1M2, VTI1B, EXOC5, CLTC, STEAP2 | 0.0216 |
| GO:0003006 reproductive developmental process | 12 | ACVR2A, CCND1, FGF7, HOXA13, DMRT1, AFF4, TGFB3, IDH1, EIF2B2, FNDC3A, TBPL1, GJB2 | 0.0243 |
| GO:0007389 pattern specification process | 12 | ACVR2A, DAND5, FKBP8, SATB2, DLX1, ARC, TSHZ1, SOSTDC1, CYP26B1, GDF11, AXIN2, RTTN | 0.0274 |
| GO:0060021 palate development | 4 | SATB2, TSHZ1, TGFB3, TGFBR3 | 0.0298 |
| GO:0008406 gonad development | 7 | ACVR2A, CCND1, FGF7, DMRT1, IDH1, EIF2B2, FNDC3A | 0.0320 |
| GO:0006892 post-Golgi vesicle-mediated transport | 5 | AP1M2, VTI1B, EXOC5, CLTC, STEAP2 | 0.0344 |
| GO:0015813 L-glutamate transport | 3 | SLC17A8, SLC1A2, SLC1A3 | 0.0345 |
| GO:0006835 dicarboxylic acid transport | 3 | SLC1A2, SLC1A3, SLC25A10 | 0.0393 |
| GO:0015837 amine transport | 7 | SLC36A1, SLC17A8, SLC1A2, SLC1A3, PDPN, CTNS, SLC25A15 | 0.0397 |
| GO:0016049 cell growth | 5 | ATP6V0E2, TGFB3, TGFBR3, DCX, VAT1 | 0.0403 |
| GO:0016567 protein ubiquitination | 7 | RNF8, SUZ12, RNF144B, WWP1, UBE4B, RNF217, FBXO10 | 0.0411 |
| GO:0040007 growth | 9 | SLC1A2, ATP6V0E2, TAF8, GDF11, TGFB3, TGFBR3, ICMT, DCX, VAT1 | 0.0415 |
| GO:0032989 cellular component morphogenesis | 15 | DNM1L, PDPN, DRD2, ONECUT2, KIF5C, PRKCI, CUL3, SLC1A3, TGFBR3, MAP7, CNTN4, CLASP2, SEMA3A, DCX, TBPL1 | 0.0441 |
| GO:0015800 acidic amino acid transport | 3 | SLC17A8, SLC1A2, SLC1A3 | 0.0443 |
| GO:0006536 glutamate metabolic process | 3 | SLC1A3, GLUD2, GLUD1 | 0.0443 |
| GO:0030512 negative regulation of transforming growth factor beta receptor signaling pathway | 3 | DAND5, ONECUT2, TGFB3 | 0.0495 |
| GO:0042787 protein ubiquitination during ubiquitin-dependent protein catabolic process | 3 | RNF144B, UBE4B, RNF217 | 0.0495 |
